# Proximity-Based Phospho-Interactome (Prob-PhI) Characterization Reveals Distinct Signaling Activities of MEK1 and MEK2

**DOI:** 10.1021/acs.analchem.5c05557

**Published:** 2026-01-29

**Authors:** Ying Wang, Ping Xiao, Ligang Fan, Yilin Pan, Haiying Ma, Rui Wang, Cuixiang Xu, Liang Zhang

**Affiliations:** 1 Shaanxi Provincial Key Laboratory of Infection and Immune Diseases, 159431 Shaanxi Provincial People’s Hospital, Xi’an 710068, China; 2 Shaanxi Engineering Research Center of Cell Immunology, 159431 Shaanxi Provincial People’s Hospital, Xi’an 710068, China; 3 Department of Biomedical Sciences, College of Veterinary Medicine and Life Sciences, 53025 City University of Hong Kong, 83 Tat Chee Avenue, Kowloon, Hong Kong China; 4 College of Biological Science and Food Engineering, 12617 Southwest Forestry University, Kunming 650224, China; 5 Ministry of Education Key Laboratory of Resource Biology and Biotechnology in Western China; Shaanxi Provincial Key Laboratory of Biotechnology; School of Medicine, 12657 Northwest University, Xi’an 710069, China; 6 Shenzhen Bay Laboratory, PKU-Princeton Research Center for Drug Development, 5F No.9 Duxue Rd. Nanshan District, Shenzhen 518000, China; 7 Key Laboratory of Biochip Technology, Biotech and Health Centre, Shenzhen Research Institute of City University of Hong Kong, Shenzhen 518057, China

## Abstract

Protein kinases play a key role in regulating cellular processes through protein phosphorylation. Comprehensive identification of kinase-specific substrates is essential for elucidating mechanisms of health and disease, yet remains a significant challenge. Here, we present the proximity-based phospho-interactome (Prob-PhI) platform—a novel and streamlined method for dissecting kinase interactomes and substrate profiles. Prob-PhI utilizes the rapid biotin ligase BASU to label proteins in proximity to a kinase of interest. Phosphorylation events among these biotinylated interactors are then enriched and analyzed under conditions with and without kinase inhibition, enabling the identification of differential phosphorylation and corresponding substrates. We applied Prob-PhI to MEK1 and MEK2, central components of the mitogen-activated protein kinase (MAPK) pathway, and delineated their distinct interactomes and phosphoproteomes. Notably, functional validation revealed that MEK2, but not MEK1, specifically interacts with and phosphorylates lysosome-associated membrane glycoprotein 3 (LAMP3) at threonine 201, thereby modulating lysosomal function. This study highlights the unique substrate profiles of MEK1 and MEK2 and demonstrates the applicability of Prob-PhI in mapping kinase signaling networks.

## Graphical abstract

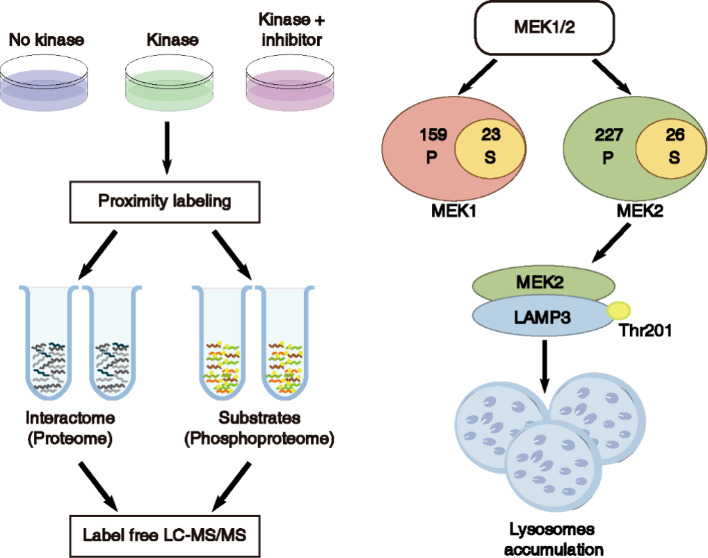

## Introduction

Protein kinases are central regulators of cellular signaling pathways, orchestrating key physiological processes such as proliferation, differentiation, and metabolism through the phosphorylation of specific target proteins.^1^ Identifying kinase substrates remains a complex challenge, requiring integrated experimental strategies to capture phosphorylation events and define kinase-substrate relationships. Existing approaches offer complementary strengths but also face notable limitations. For example, phosphoproteomics, which employs mass spectrometry (MS) to compare phosphopeptide profiles under differential kinase activity, provides high sensitivity but may overlook low-abundance substrates.^2^ In parallel, kinase substrate profiling arrays enable rapid screening using predefined sets of peptides and antibodies, yet are constrained by limited scope.^3^ Substrate trapping, which uses engineered kinase mutants to stabilize transient interactions, facilitates mechanistic insights but demands precise mutant design.^4^ Moreover, integrating chemical proteomics and genetic approaches with bioinformatics tools offers functional insights but requires rigorous validation.^5–7^

Recently, proximity labeling has emerged as a powerful strategy to address these challenges in mapping kinase-substrate networks.^8,9^ Proximity labeling systems primarily fall into two categories: engineered biotin ligases and peroxidases. First-generation ligases like BioID offer low cytotoxicity but require long labeling times (18–24 h), limiting their ability to capture dynamic interactions. Second-generation variants such as TurboID and miniTurbo achieve faster labeling (minutes), but their high activity often causes background biotinylation and cellular toxicity by depleting essential biotin cofactors, potentially disrupting native signaling pathways. In contrast, BASU—an engineered ligase derived from *Bacillus subtilis*—combines rapid labeling kinetics with low toxicity. Its compact size (∼29 kDa) minimizes steric hindrance, and its mechanism avoids the metabolic disruption seen with TurboID, making it well-suited for phosphoproteomic applications.^10^ Peroxidase-based systems (e.g., APEX/APEX2) also enable fast labeling but require cytotoxic hydrogen peroxide, which induces oxidative stress and interferes with phosphorylation signaling dynamics. Recent efforts have combined proximity labeling with chemical proteomics to investigate kinase interactomes and substrate profiles. For example, Niinae et al. integrated BioID-based labeling with kinase perturbation phosphoproteomics and motif analysis to predict substrates of casein kinase 2 (CK2) and protein kinase A (PKA).^11^ Similarly, BioID2 data mining has been used to identify the p38α interactome.^12^ While these studies have yielded valuable insights, they often rely on separate experimental and computational pipelines to analyze interactors and substrates—an approach that may obscure functional relationships. An optimized strategy would integrate proximity labeling, functional proteomics, and quantitative phosphoproteomics into a unified workflow, enabling simultaneous resolution of kinase-substrate pairs within broader interaction networks.

Among protein kinases, MEK1 and MEK2—dual-specificity kinases within the mitogen-activated protein kinase (MAPK) family—play pivotal roles in regulating cell proliferation, differentiation, and survival.^13^ These kinases share extensive structural homology, including conserved domains in various functional aspects.^14^ Despite their similarities, accumulating evidence points to distinct functional roles. For example, MEK2 knockout mice are viable and develop normally,^15^ whereas MEK1 deficiency leads to embryonic lethality,^16^ highlighting their nonredundant biological functions. At the cellular level, MEK2 depletion alters morphology and impairs cancer cell invasiveness, while MEK1 knockdown primarily inhibits proliferation via G0/G1 cell cycle arrest.^17^ These findings suggest that MEK1 and MEK2, though structurally analogous, exert specialized and nonoverlapping roles in cellular physiology and oncogenesis. To elucidate the functional divergence between MEK1 and MEK2, a comprehensive characterization of their interactomes, substrate profiles, and regulated signaling pathways is essential. However, a systematic and comparative analysis of MEK1/2 interaction networks and phosphorylation substrates remains lacking.

To address this gap, we developed Proximity-based Phospho-Interactome Mapping (Prob-PhI)–an integrated platform that simultaneously captures kinase interactors and substrates within a streamlined workflow (Scheme 1). Prob-PhI employs the engineered biotin ligase BASU, derived from *Bacillus subtilis* BirA*, which exhibits >1,000-fold faster biotinylation kinetics than conventional *E. coli* BirA* systems. This enhanced efficiency enables the capture of transient kinase-substrate interactions within biologically relevant 5 min labeling windows, while avoiding the cytotoxicity associated with APEX-based methods. By analyzing biotinylated interactors and their phosphorylation states in the presence and absence of the MEK1/2 inhibitor trametinib,^18^ we identified differential phosphorylation patterns that revealed distinct substrate profiles for MEK1 and MEK2. Prob-PhI thus provides a high-confidence, integrated view of MEK1/2 interactomes and phosphoproteomes, offering new insights into their divergent signaling activities.

**Scheme 1:**
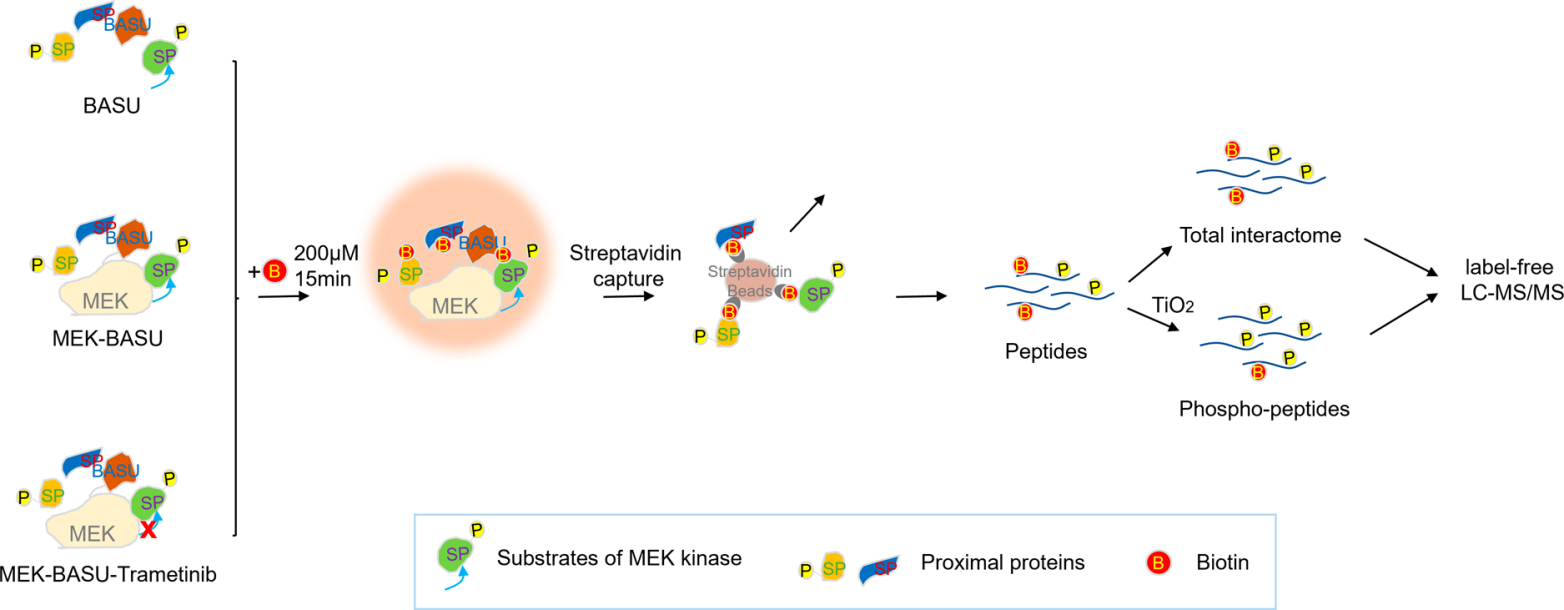
Schematic Outline of Prob-PhI for MEK Kinase Interactome and Phosphoproteome Analysis

## Experimental Methods

### Cell Culture, Transfection, and Treatments

HEK293T or HeLa cells were seeded in 100 mm culture dishes and transfected at ∼70% confluence using PEI (Polysciences, 23966) with plasmids encoding BASU, MEK1-BASU, or MEK2-BASU. After 24 h, one set of MEK1-BASU and MEK2-BASU transfected cells was treated with 1 μM trametinib (MEK inhibitor; MCE, HY-10999) for an additional 24 h. Each experimental group consisted of five 100 mm dishes. At 48 h post-transfection, cells were incubated with 200 μM biotin (Sigma, B4639) for 15 min at 37 °C before harvest by centrifugation and washed three times with cold PBS. Pellets were lysed in ice-cold TNTE buffer (50 mM Tris-HCl, pH 7.4; 150 mM NaCl; 1% Triton X-100; 1 mM EDTA) supplemented with protease and phosphatase inhibitors.

### Isolation of Biotinylated Proteins, Digestion and Phosphopeptide Enrichment

Biotinylated proteins were enriched using streptavidin magnetic beads (MCE, HY-K0208) with 2-h incubation at 4 °C under rotation. Beads were washed five times with Buffer II (PBS, pH 7.4, 0.05% Tween-20). For on-bead digestion, beads were resuspended in 250 μL of Elution Buffer I (50 mM Tris-HCl, pH 8.0; 2 M urea; 10 μg/mL sequencing-grade trypsin (Thermo Fisher, 90058); 1 mM DTT) and incubated at 30 °C, 400 rpm for 60 min. Supernatants were collected via magnetic separation.

Beads were then resuspended twice in 125 μL of Elution Buffer II (50 mM Tris-HCl, pH 8.0; 2 M urea; 5 mM iodoacetamide), protected from light. Supernatants from all elution steps were pooled, and an additional 500 ng of trypsin was added. Samples were incubated overnight at 32 °C, 400 rpm, protected from light. Digestion was quenched by adding 10% formic acid (FA) at a 1:25 (v/v) ratio. Peptides were desalted using pre-equilibrated C18 SepPak cartridges prior to phosphopeptide enrichment.

Phosphopeptides were enriched using Titansphere Phos-TiO tip columns (GL Sciences, 5010–21308). Peptides were dissolved in 0.1% FA and incubated with the tips for 10 min at room temperature. Tips were washed twice with Buffer B (100% acetonitrile, 2% TFA), followed by two washes with Buffer A (100% acetonitrile, 2% TFA, DL-lactic acid). Phosphorylated peptides were eluted with 50 μL of 5% ammonium hydroxide, followed by a wash with 5% pyrrolidine. Samples were vacuum-dried and stored at – 80 °C until LC-MS/MS analysis.

### Mass Spectrometry and Data Processing

Peptide samples were reconstituted in 20 μL of 0.1% FA and analyzed using an Easy-nLC 1200 system (Thermo Scientific) coupled to a Q-Exactive HF Orbitrap mass spectrometer (Thermo Scientific). Peptides were loaded onto a reverse-phase C18 column (75 μm × 15 cm, 3 μm particle size) and eluted over a 75 min linear gradient (7–95% buffer B: 0.1% FA in 80% acetonitrile) at 250 nL/min.

Samples were analyzed in positive ion mode. Survey scans were acquired at 120,000 resolutions over an *m*/*z* range of 350–1800, with an AGC target of 3 × 10^6^. The top 12 most intense ions were selected for HCD fragmentation (normalized collision energy: 27), and MS/MS spectra were acquired at 30,000 resolutions with an AGC target of 1 × 10^5^. Dynamic exclusion was set to 30 s.

Raw MS/MS data were processed using Sequest HT within Proteome Discoverer (PD) v2.2 (Thermo Scientific). Precursor and fragment mass tolerances were set to 10 ppm and 0.02 Da, respectively. Up to two missed cleavages were allowed. Carbamidomethylation (C) was set as a fixed modification; oxidation (M), phosphorylation (S/T/Y), and N-terminal acetylation were set as variable modifications. Peptide spectrum matches (PSMs) were filtered at a 1% false discovery rate (FDR) using the Percolator algorithm. Label-free quantification was performed using precursor ion areas via Minora Feature alignment in PD 2.2.

### Module Clustering and Enrichment Analysis

MEK-interacting proteins were clustered using the fuzzy C-means algorithm from the Mfuzz R package,^19^ with the number of clusters set to 2. Each cluster was analyzed for protein–protein interactions (PPIs) using STRING (http://string-db.org) and annotated via the KEGG pathway database (https://www.genome.jp/kegg/pathway.html) using a combined score >0.4 and FDR ≤ 0.05.

Phosphorylated proteins were clustered into four groups using the same algorithm. Functional enrichment was performed using Metascape,^20^ which integrates multiple ontology resources. PPI networks were constructed with a combined score >0.4, and representative enriched terms were visualized using Cytoscape v3.9.0.

### Phosphosite Motif Analysis

To identify amino acid motifs flanking phosphorylated (S/T) residues, the MOMO tool from the MEME Suite v5.5.5 was used with the motif-x algorithm^21,22^ applying a motif width of 9 amino acids.

## Results

### Establishment and Validation of the Proximity-Based Phospho-Interactome (Prob-PhI) System

To systematically dissect the interactome and substrate profiles of MEK1 and MEK2, we developed the Proximity-based Phospho-Interactome (Prob-PhI) workflow (Figure 1A). This platform integrates proximity labeling with phosphoproteomic analysis to capture kinase-associated proteins and phosphorylation events in a single streamlined approach. We generated expression constructs encoding MEK1 or MEK2 fused to the fast-acting biotin ligase BASU, along with an HA tag and EGFP reporter linked via a self-cleaving T2A peptide (Figure 1B). The superior kinetics of the BASU were validated in a direct comparison with BirA*, a common biotin ligase used in BioID (Figure S1A). A control construct expressing BASU alone was used to define background biotinylation levels. To identify kinase substrates, phosphopeptides from biotinylated interactors were enriched and analyzed by mass spectrometry (MS), with and without treatment using trametinib—a selective MEK inhibitor.

**Figure 1:**
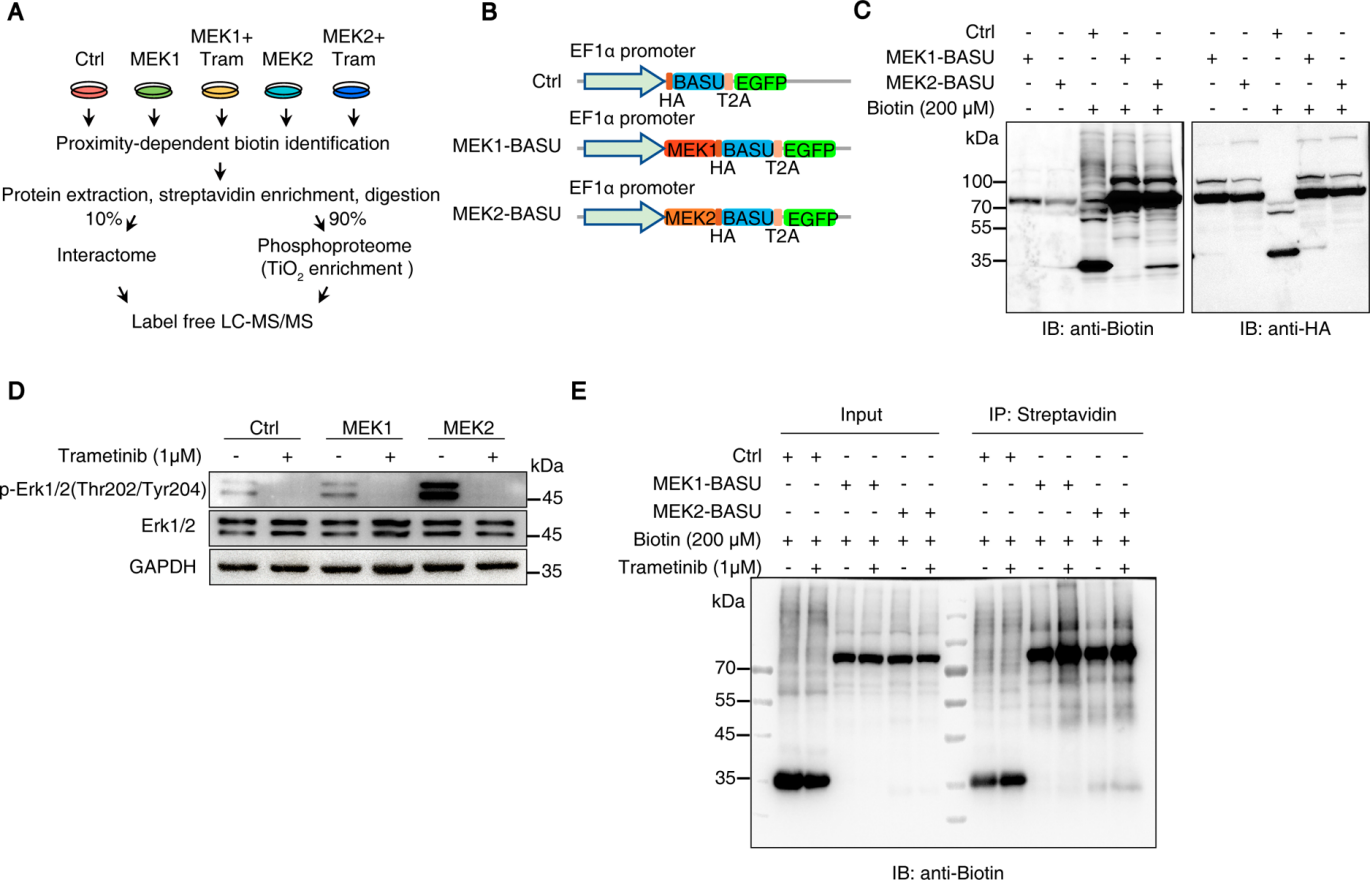
The Prob-PhI system was well established. (A) Schematic outline of the MEK kinase interactome and phosphoproteome analysis. (B) Plasmids used in this study. HA: hemagglutinin tag; T2A: T2A self-cleaving peptide; EGFP: enhanced green fluorescent protein. (C) HEK293T cells were transfected with BASU, MEK1-BASU, and MEK2-BASU for 48 h. After treatment with 200 μM biotin for 15 min, cell lysates were collected, and Western blotting was performed using anti-biotin and anti-HA primary antibodies. (D) and (E) HEK293T cells were transfected with Ctrl (BASU), MEK1-BASU, and MEK2-BASU for 24 h and then treated with trametinib (a MEK1/2 inhibitor) for another 24 h. After treatment with 200 μM biotin for 15 min, cell lysates were collected for streptavidin pull-down assays, prior to immunoblotting to detect p-ERK1/2, t-ERK1/2, and biotinylated proteins as indicated.

Upon transfection into HEK293T cells, comparable HA expression levels were observed across all experimental groups, confirming consistent construct expression (Figure 1C, right panel). Following biotin treatment, distinct biotinylation profiles were detected in cells expressing BASU, BASU-MEK1, and BASU-MEK2 (Figure 1C, left panel), indicating differential proximal protein labeling and suggesting unique interactomes for MEK1 and MEK2.

To validate the ability of Prob-PhI to detect MEK1/2 regulated phosphorylation events, we treated cells with trametinib and confirmed reduced phosphorylation of ERK1/2, canonical downstream effectors of MEK signaling (Figure 1D). Although trametinib treatment did not markedly alter the overall biotinylation levels of MEK1/2-associated proteins in whole-cell lysates, it enhanced the enrichment of specific biotinylated proteins compared to untreated controls (Figure 1E). These findings support the robustness of the Prob-PhI system in capturing kinase-specific interactions and phosphorylation dynamics within cellular contexts.

### Defining MEK1/2 Interactome Using Prob-PhI

Following expression of MEK1/MEK2-BASU fusion constructs and biotin treatment, biotinylated proteins were enriched using streptavidin beads, digested into peptides, and analyzed by LC-MS/MS with label-free quantification. For downstream analysis, 10% of the sample was allocated for interactome profiling, while the remaining 90% was used for phosphoproteome investigation (Figure 1A). Principal component analysis (PCA) of the interactome data revealed clear separation between experimental conditions, with replicates clustering tightly indicating high reproducibility (Supplementary Figure S1B).

In total, ∼2,500 proteins were quantified from streptavidin pulldowns (Figure 2A; Supplementary Table S1). Trametinib treatment did not significantly alter the number of biotinylated proteins, consistent with Western blot results (Figure 1E). To identify MEK1/MEK2-specific interactors, we performed differential analysis comparing MEK1/MEK2-overexpressing groups to the BASU-only control, using a cutoff of *p* < 0.01 and log_2_ fold change >2. Among the enriched proteins, known MEK1/MEK2 interactors such as NRAS and CSK were identified (highlighted in orange in Figure 2B and 2C), validating the specificity and robustness of the Prob-PhI platform. In total, 182 proteins were identified as MEK1 interactors and 253 as MEK2 interactors, with 90 proteins shared between both groups (Figure 2D; Supplementary Table S2), indicating both overlap and divergence in their interactomes.

**Figure 2:**
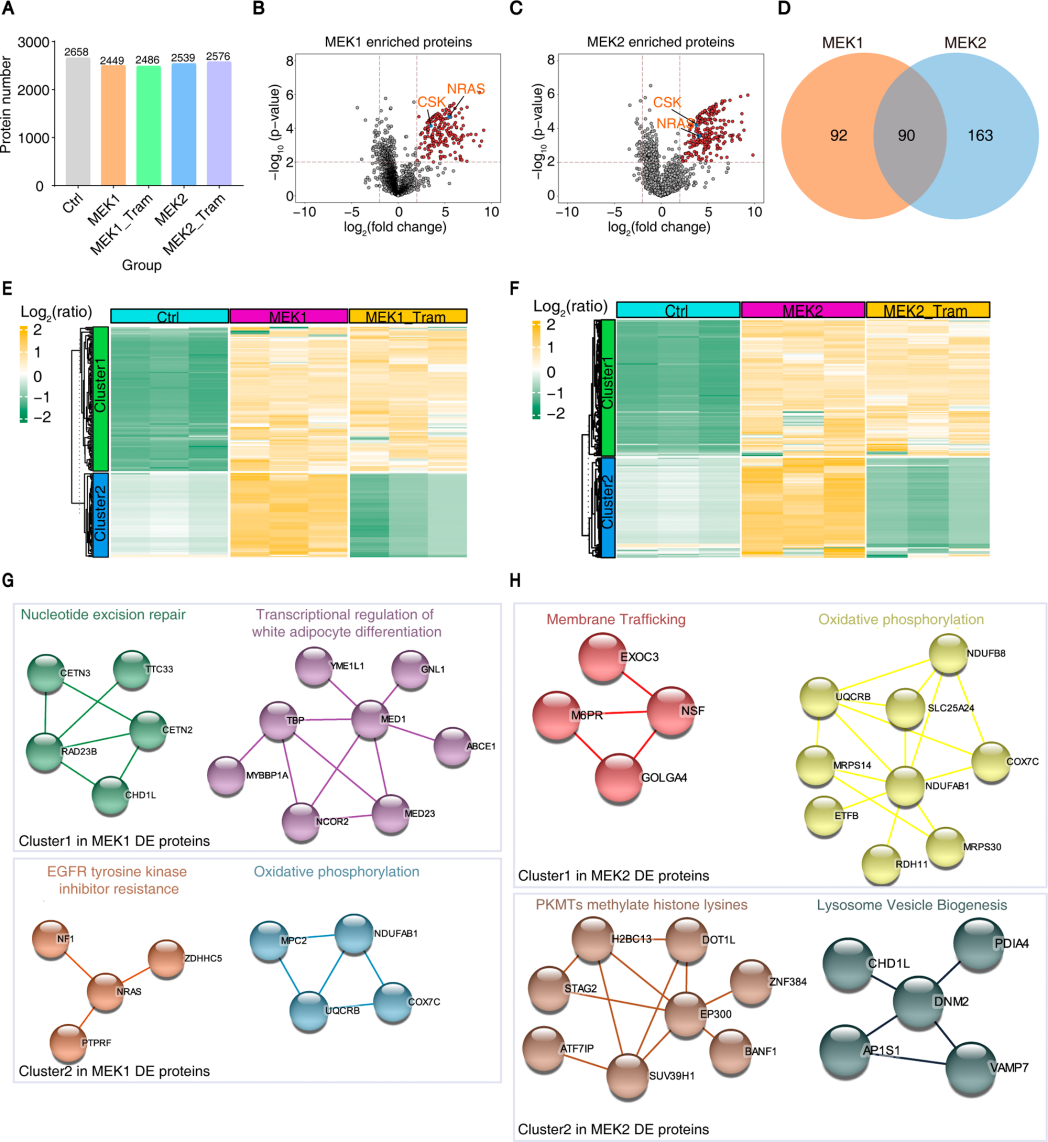
Characterization of the MEK1- and MEK2-regulated proteome. (A) The bar chart illustrates the number of proteins identified in different experimental groups. (B) Volcano plot of MEK1-associated proteins identified by Pro-PhI. Colors reflect proteins with significant changes in MEK1 group (up: red) using the thresholds of log_2_(fold change) > 2 and *p*-value <0.01. (C) Volcano plot of MEK2-associated proteins identified by Pro-PhI. Colors reflect proteins with significant changes in MEK2 group (up: red) using the thresholds of log_2_(fold change) > 2 and *p*-value <0.01. (D) Venn diagram showing the number of candidate interacting proteins that overlap between the MEK1/Ctrl and MEK2/Ctrl groups (log_2_(fold change) > 2 and *p*-value <0.01). (E) Clustering analysis of MEK1-associated proteins, which were stratified into trametinib-insensitive (Cluster 1) and trametinib-sensitive (Cluster 2) groups. (F) Clustering analysis of MEK2-associated proteins, which were stratified into trametinib-insensitive (Cluster 1) and trametinib-sensitive (Cluster 2) groups. (G) Functional enrichment analysis of the trametinib-insensitive interactors (Cluster 1) and trametinib-sensitive interactors (Cluster 2) of MEK1. (H) Functional enrichment analysis of the trametinib-insensitive interactors (Cluster 1) and trametinib-sensitive interactors (Cluster 2) of MEK2.

To assess the impact of trametinib on MEK1/MEK2 interactions, clustering analysis was performed, revealing two distinct clusters (Figure 2E,F). Cluster 1 comprised proteins whose interactions were largely unaffected by trametinib, suggesting kinase activity-independent associations. In contrast, Cluster 2 included proteins whose interactions were markedly reduced upon trametinib treatment, indicating phosphorylation-dependent binding. Notably, volcano plot visualization showed that Cluster 2 proteins exhibited higher enrichment relative to controls compared to Cluster 1 (Supplementary Figure S1C), suggesting that phosphorylation-dependent interactions are more dynamic and efficiently labeled within the short biotinylation window.

To explore the functional relevance of MEK1/MEK2 interactomes, we mapped the clustered proteins onto STRING protein–protein interaction networks. For MEK1 Cluster 1, functional modules related to nucleotide excision repair and transcriptional regulation of white adipocyte differentiation were identified (Figure 2G, top), suggesting roles beyond canonical MAPK signaling. MEK1 Cluster 2 revealed a NRAS-centered complex associated with EGFR tyrosine kinase inhibitor resistance and a module linked to oxidative phosphorylation (Figure 2G, bottom), indicating trametinib-sensitive interactions with potential therapeutic relevance.

For MEK2 Cluster 1, modules involved in membrane trafficking and oxidative phosphorylation (centered on NDUFAB1) were observed (Figure 2H, top). Although both MEK1 and MEK2 interact with oxidative phosphorylation components, their distinct clustering patterns suggest specialized roles. MEK2 Cluster 2 revealed an EP300-centered complex involved in histone methylation and a dynamin 2-centered module linked to late endosome/lysosome biogenesis (Figure 2H, bottom), highlighting MEK2’s potential role in cellular quality control and sensitivity to trametinib.

### Phosphoproteomic Profiling of MEK1 and MEK2 Regulated Proteins

In addition to interactome mapping, 90% of the Prob-PhI samples were used to identify kinase substrates by examining phosphorylation sites sensitive to MEK1/2 inhibition. Phosphopeptides were enriched using TiO_2_ and analyzed via label-free LC-MS/MS (Figure 1A). In total, we identified 4,059 and 4,286 phosphopeptides corresponding to 1,133 and 1,173 parent proteins in MEK1- and MEK2-expressing cells, respectively (Supplementary Figure S2A–B; Supplementary Table S3). Tight clustering of sample groups confirmed high technical reproducibility and the discriminative power of Prob-PhI (Supplementary Figure S2C).

To characterize phosphorylation dynamics regulated by MEK1 and MEK2, we performed fuzzy C-means clustering^23^ on significantly enriched phosphosites across control, MEK, and MEK + trametinib conditions. This analysis revealed four distinct clusters for both MEK1 and MEK2 (Figure 3A,B; Supplementary Table S4), representing unique phosphorylation patterns: Cluster 1: Upregulated by MEK expression, attenuated by trametinib; Cluster 2: Maintained under MEK expression, reduced by trametinib; Cluster 3: Downregulated by MEK, sustained with trametinib; and Cluster 4: Upregulated and maintained despite trametinib. The similarity in clustering patterns between MEK1 and MEK2 suggests potential shared substrates or cooperative signaling mechanisms.

**Figure 3:**
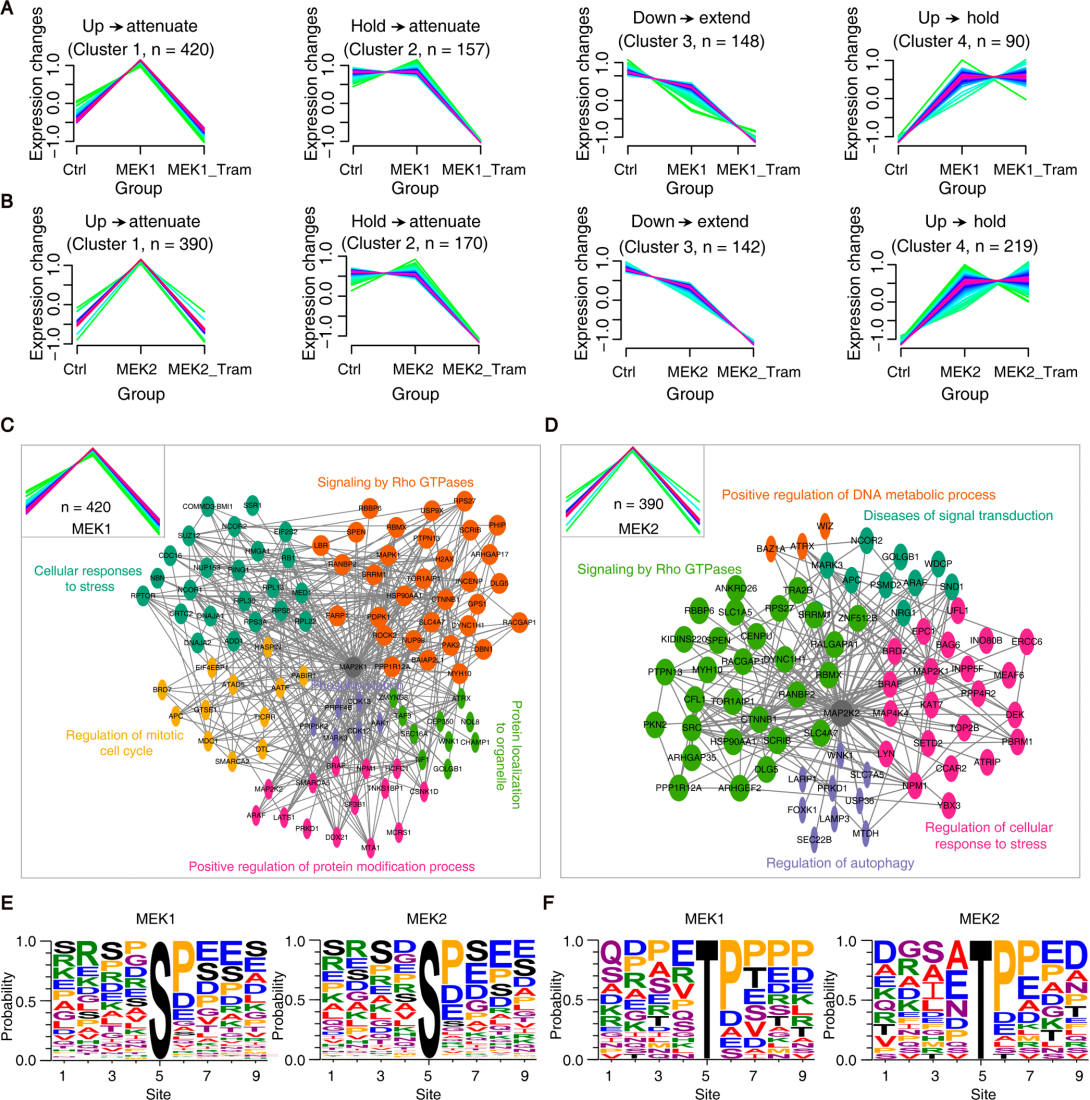
Phosphoproteome profiling reveals expression clusters and functional modules regulated by MEK1 and MEK2. (A) Four clusters of MEK1-enriched phosphosites identified by Fuzzy C-Means clustering analysis. (B) Four clusters of MEK2-enriched phosphosites identified by Fuzzy C-Means clustering analysis. (C,D) Protein–protein interaction network and gene ontology (GO) enrichment analyses were performed for Cluster 1 of MEK1- and MEK2-enriched phosphosites using Metascape, respectively. (E,F) Phosphorylation motif analysis was performed by MoMo, utilizing a 9-amino-acid motif window centered on the phosphorylation site within Cluster 1 of MEK1- and MEK2-enriched phosphosites; the frequency of each amino acid at each position was quantified to generate motif logos.

Cluster 1 was the largest, encompassing hundreds of phosphosites that were induced by MEK1/2 and suppressed by trametinib–consistent with direct kinase-substrate relationships. Notably, this cluster included known MEK substrates such as ERK2 phosphorylated at tyrosine 187 (Y187),^24^ validating the specificity of Prob-PhI. Gene ontology (GO) enrichment analysis of Cluster 1 parent proteins revealed distinct functional profiles. MEK1-regulated proteins were enriched in cell cycle regulation, Rho GTPase signaling, stress response, protein modification, and organelle localization (Figure 3C). MEK2-regulated proteins showed enrichment in signal transduction disorders, Rho GTPase regulation, stress response, and autophagy (Figure 3D). These findings highlight the biological relevance of MEK1/2-mediated phosphorylation in diverse cellular processes.

To further investigate substrate recognition motifs, we applied the MoMo algorithm^25^ to analyze amino acid sequences flanking the phosphosites in Cluster 1 using a 9-residue window. Motif analysis revealed a strong preference for proline at the +1 position (Figure 3E), and frequent occurrence of glutamic acid and proline at +3 (Figure 3F). These conserved features suggest a role in substrate specificity and phosphorylation efficiency, offering mechanistic insights into MEK1/2 signaling regulation.

To integrate phosphorylation dynamics with protein interactions, we cross-referenced the phosphoproteome data (Figure 3C,D) with the interactome profiles (Figure 2D). This analysis identified 23 MEK1 and 26 MEK2 interactors that exhibited trametinib-sensitive phosphorylation events—representing high-confidence candidate substrates (Figure 4A,B). Comparative analysis revealed 17 unique substrates for MEK1 and 20 unique substrates for MEK2, with 6 shared substrates between both kinases (Figure 4C). Heatmap visualization of these phosphopeptides demonstrated clear induction upon MEK1/2 overexpression and attenuation following trametinib treatment (Figure 4D,E), consistent with direct kinase-substrate relationships. These findings further validate the utility of Prob-PhI in identifying functional substrates within kinase interactomes.

**Figure 4:**
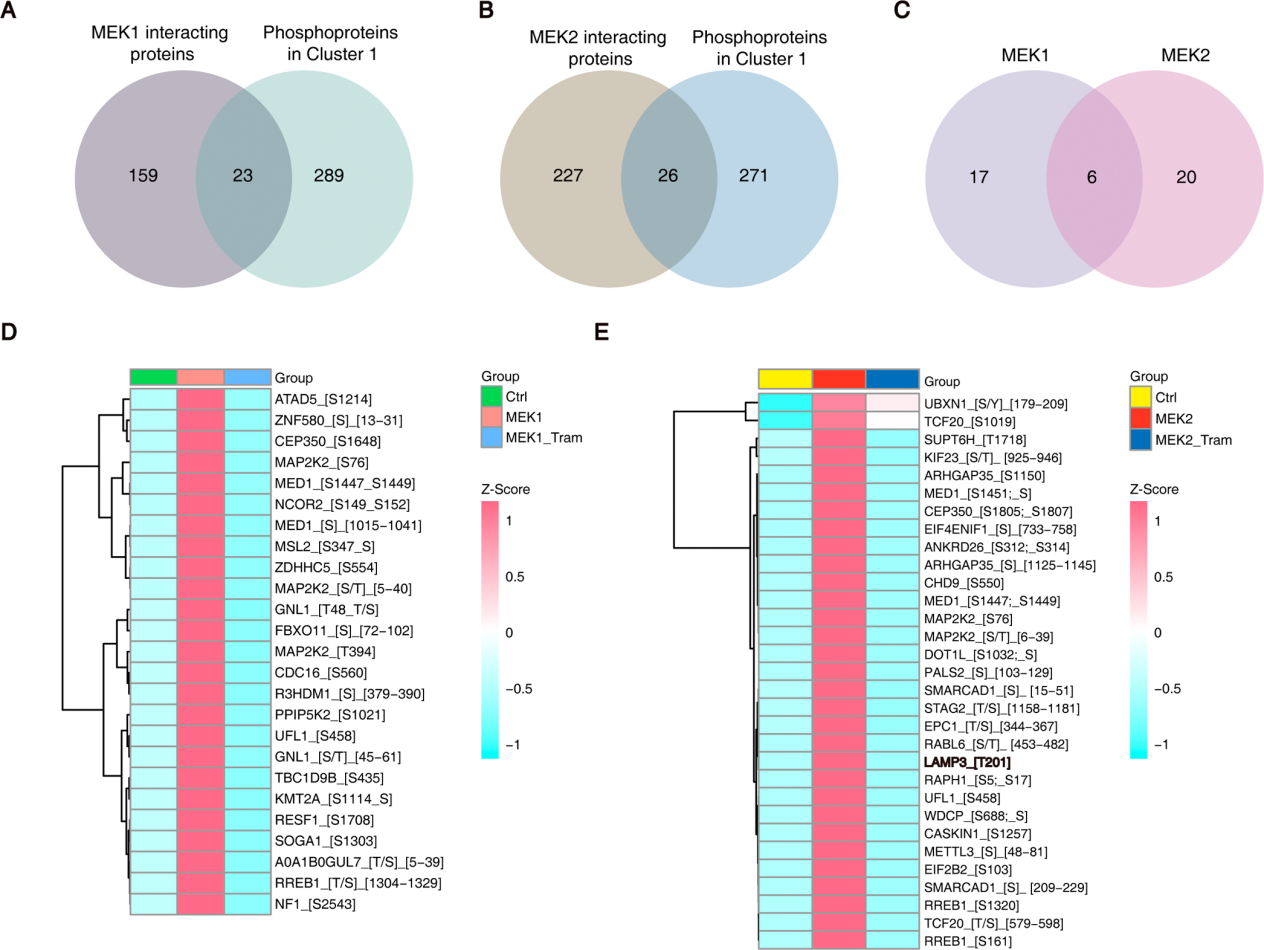
Characterization of candidate substrates of MEK1 and MEK2. (A) Venn diagram illustrated the overlapping between the proteins that are significantly enriched in the MEK1 interactome and the phosphoproteins identified in Cluster 1 of the MEK1 group. (B) Venn diagram showed the overlapping between the proteins that are significantly enriched in the MEK2 interactome and the phosphoproteins identified in Cluster 1 of the MEK2 group. (C) The Venn diagram was employed to depict the overlap between the substrates that are significantly enriched in MEK1 and MEK2 interactomes. (D) The heatmap visualized the expression levels of candidate substrate proteins for MEK1 in Ctrl, MEK1-OE, MEK1-OE with trametinib. Each row in the heatmap represented a candidate substrate protein, and the color intensity represents the expression level, with red color indicating higher expression. (E) The heatmap depicted the expression levels of candidate substrate proteins for MEK2 in Ctrl, MEK2-OE, MEK2-OE with trametinib. Each row in the heatmap represented a candidate substrate protein, and the color intensity reflected the expression level, with red color indicating higher expression.

Among the differential substrates, LAMP3 emerged as a MEK2-specific interactor phosphorylated at threonine 201—a previously unreported site. This phosphorylation event, coupled with LAMP3′s selective interaction with MEK2, highlights its potential role as a novel MEK2 substrate with functional relevance.

### MEK2 Physically Interacted with LAMP3

Our phosphoproteomic and interactome analyses suggested that LAMP3 is a substrate of MEK2, but not MEK1. To validate this, we expressed Flag-tagged MEK1 or MEK2 in HEK293T cells and performed anti-Flag immunoprecipitation (IP). MEK2, but not MEK1, successfully pulled down endogenous LAMP3 (Figure 5A), consistent with our mass spectrometry findings. Furthermore, IP of endogenous MEK2 confirmed its interaction with LAMP3 in HEK293T cells (Figure 5B), reinforcing the specificity of the MEK2–LAMP3 association.

**Figure 5:**
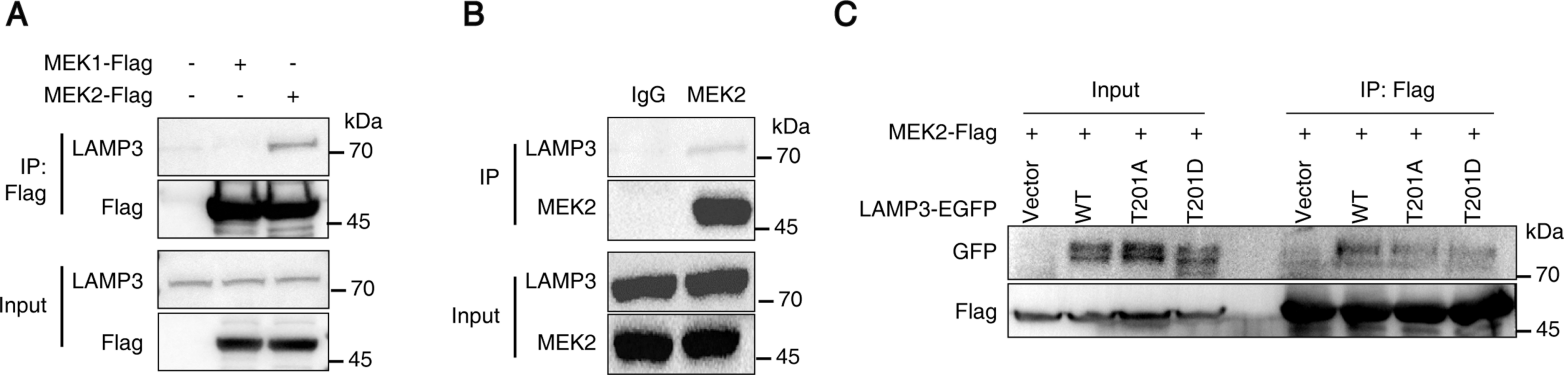
A physical interaction between MEK2 and LAMP3. (A) HEK293T cells were transfected with pcDNA-Flag, MEK1-Flag, and MEK2-Flag plasmids for 48 h. Following transfection, cell lysates were collected, and immunoprecipitation was performed using Flag tag beads. The immunoprecipitated samples were then subjected to Western blot analysis using antibodies against LAMP3 and the Flag tag to detect the presence of MEK1 and MEK2. (B) Co-immunoprecipitation was performed in HEK293T cell lysates. The lysates were incubated with a primary antibody against MEK2, followed by pulldown using protein A/G beads. The immunoprecipitated samples were then analyzed by Western blotting, probing for both LAMP3 and MEK2. IgG antibody as a control for nonspecific binding was included. (C) Co-immunoprecipitation assays were performed in HEK293T cells coexpressing MEK2 and either wild-type (WT) LAMP3, phospho-abolishing (T201A), or phospho-mimetic (T201D) LAMP3 mutants. Representative Western blots of immunoprecipitate (anti-FLAG for MEK2) and whole cell lysates (Input) probed with the indicated antibodies.

To assess the role of T201 phosphorylation in the MEK2–LAMP3 interaction, we generated phospho-abolishing (T201A) and phospho-mimetic (T201D) LAMP3 mutants. Co-immunoprecipitation assays revealed that neither mutation significantly affected the interaction between LAMP3 and MEK2 (Figure 5C), suggesting that phosphorylation at T201 is not required for complex formation. These results indicate that while MEK2 phosphorylates LAMP3 at T201, the interaction itself is phosphorylation-independent.

### MEK2 Modulates LAMP3 to Promote Functional Lysosomes

To investigate the biological function of the MEK2–LAMP3 axis, we co-expressed MEK2 and LAMP3 in HEK293T cells and visualized lysosomes using Lysotracker ND99 staining. Expression of LAMP3-eGFP led to a marked increase in Lysotracker-positive vesicles localized to the perinuclear region, a phenotype absents in cells expressing eGFP alone (Figure 6A). Notably, both MEK inhibition via trametinib and phospho-abolishing mutation of LAMP3 (T201A) significantly reduced this lysosome-promoting activity (Figure 6B,C). In comparison, the phospho-mimetic T201D mutant led to lysosomal signals significantly greater than T201A, but seemingly lower than WT, though not statistically significant (Figure 6C). This suggests the importance of the dynamic phosphorylation-dephosphorylation of LAMP3 in promoting lysosomal function, which will be further investigated in future studies.

**Figure 6:**
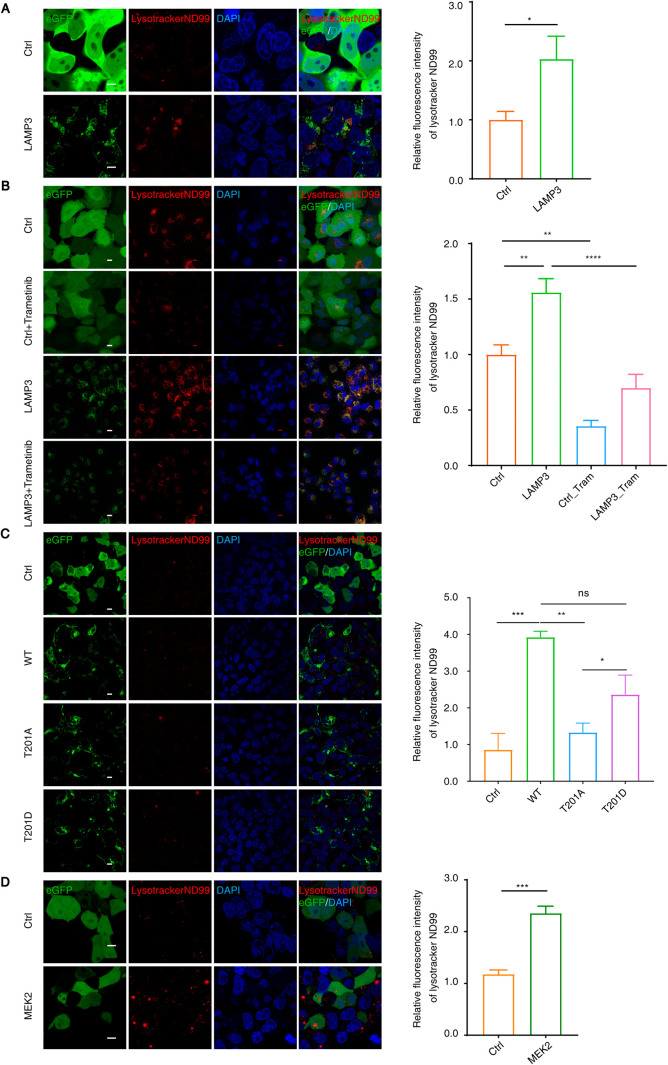
LAMP3 expressions increased the amount of functional lysosomes. (A) Immunostaining analysis was performed in HEK293T cells transfected with empty-eGFP plasmid (upper panels) or LAMP3-eGFP plasmid (lower panels). Scale bar: 10 μm. The mean fluorescence intensity of lysotracker ND99 was quantified in 100 cells per group, and the relative fluorescence intensity of lysotracker ND99 was plotted. Values are presented as mean ± SD (*n* = 3. *p < 0.05. (B) Immunostaining analysis was performed in A549 stable cell lines expressing Ctrl-eGFP or LAMP3-eGFP, with or without treatment with trametinib (1 μM) for 24 h. Scale bar: 10 μm. The mean fluorescence intensity of lysotracker ND99 was quantified in 100 cells per group, and the relative fluorescence intensity of lysotracker ND99 was plotted. Values are presented as mean ± SD (*n* = 3. ****p < 0.0001, ***p < 0.001, **p < 0.01. (C) Immunostaining analysis of Ctrl-eGFP and LAMP3^wt^-eGFP, LAMP3^T201A^-eGFP, and LAMP3^T201D^-eGFP expressed HEK293T cell lines. Scale bar: 10 μm. The mean fluorescence intensity of lysotracker ND99 in 100 cells of each group was counted, and the relative fluorescent intensity of lysotracker ND99 was plotted. Values are presented as mean ± SD (*n* = 3). ***p < 0.001, **p < 0.01, *p < 0.05, ns, not significant. (D) Immunostaining analysis of HEK293T cells transfected with empty-eGFP plasmid (upper panels) or MEK2-eGFP plasmid (lower panels) was performed. Scale bar: 10 μm. The mean fluorescence intensity of lysotracker ND99 in 100 cells of each group was counted, and the relative fluorescent intensity of lysotracker ND99 was plotted. Values are presented as mean ± SD (*n* = 3). ***p < 0.001.

Furthermore, MEK2 overexpression alone was sufficient to enhance Lysotracker signal intensity (Figure 6D), supporting its role in promoting lysosome biogenesis or activity. Collectively, these findings reveal a functional MEK2–LAMP3 signaling axis that regulates lysosomal dynamics through phosphorylation-dependent mechanisms.

### Profiling MEK1/MEK2 in HeLa Cells Using Prob-PhI

To evaluate the broader applicability of Prob-PhI, we extended the analysis to HeLa cells, a human cancer cell model. Western blot confirmed efficient biotinylation in HeLa cells (Figure 7A). Mass spectrometry analysis identified 135 MEK1 and 119 MEK2 interactors, with 14 and 9 proteins, respectively, overlapping with those found in HEK293T cells (Figure 7B; Supplementary Table S5). Most shared interactors were canonical components of the MAPK pathway (Figure 7C), supporting the conserved core signaling roles of MEK1/2. Prob-PhI also identified 35 MEK1 and 26 MEK2 phosphorylation substrates in HeLa cells, with 2 and 1 overlapping substrates shared with HEK293T, respectively (Figure 7D). These results underscore the cell-type-specific nature of MEK1/2 signaling networks. Despite differences in substrate identity, motif analysis revealed consistent phosphorylation signatures across both cell types. As in HEK293T cells, MEK1/2 substrates in HeLa cells exhibited a strong preference for proline at the +1 position, and glutamic acid or proline at +3 (Figure 7E), reinforcing the con-served substrate recognition motifs of MEK1 and MEK2.

**Figure 7:**
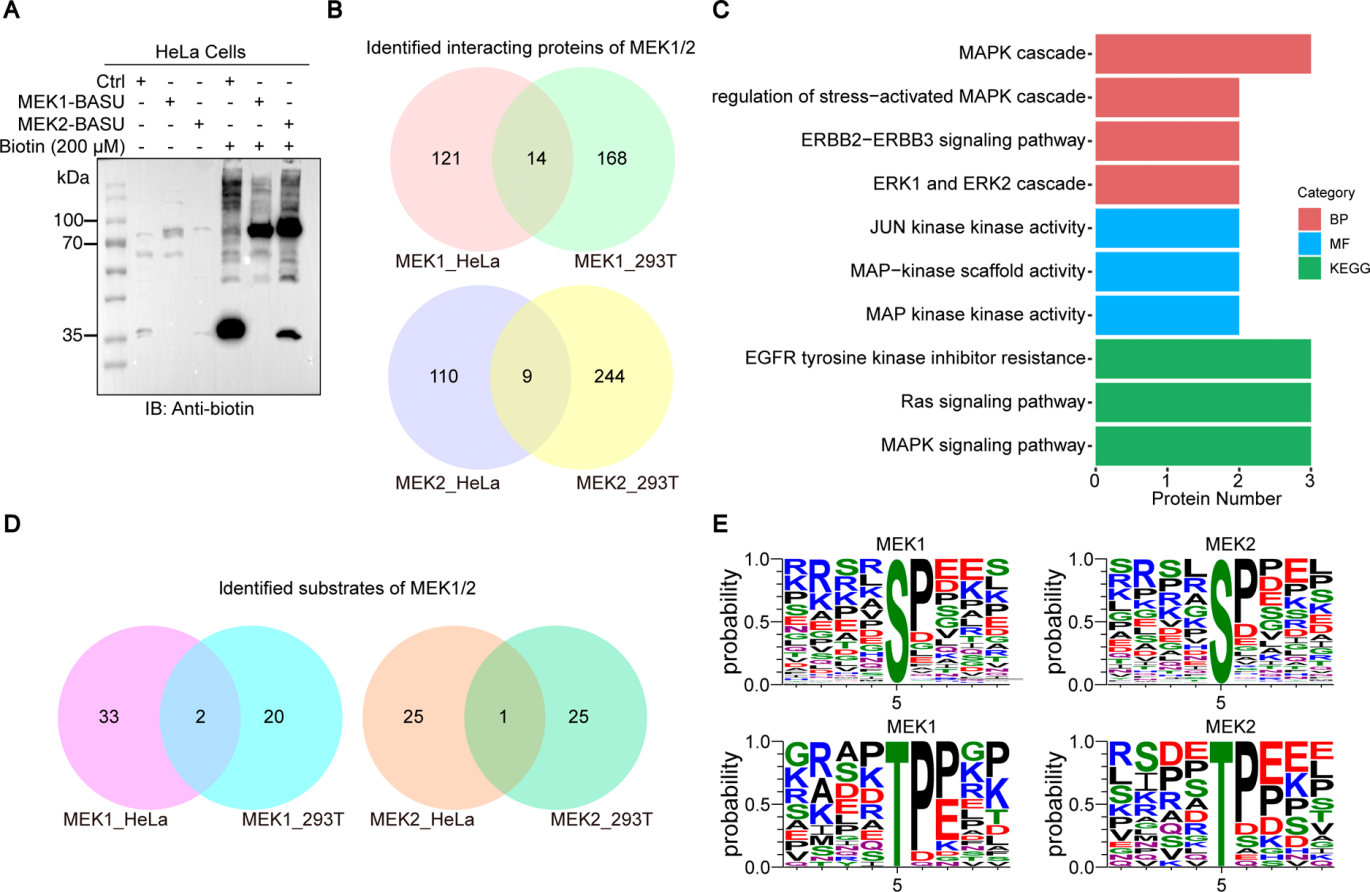
Prob-PhI is a robust and reproducible method that reveals context-specific rewiring of the MEK interactome and substrates. (A) HeLa cells were transfected with Ctrl (BASU), MEK1-BASU, and MEK2-BASU for 48 h. After treatment with 200 μM biotin for 15 min, cell lysates were collected and analyzed by Western blotting using an anti-biotin primary antibody. (B) Venn diagram showing the overlap of MEK1/2-interacting proteins identified by Prob-PhI in HeLa versus HEK293T cells. (C) Gene Ontology (GO) term analysis of the overlapping MEK1/2-interacting proteins in HeLa cells. (D) Venn diagram showing the overlap of MEK1/2 substrates identified by Prob-PhI in HeLa versus HEK293T cells. (E) Phosphorylation motif analysis for Cluster 1 of MEK1 and MEK2 enriched phosphosites in HeLa cells. Analysis was performed using MoMo, which utilized a 9-amino-acid window centered on the phosphorylation site to quantify amino acid frequency and generate motif logos.

## Conclusion and Discussion

In this study, we present the Proximity-based Phospho-Interactome (Prob-PhI) platform as a streamlined approach for kinase substrate identification and interactome profiling. Prob-PhI overcomes key limitations of conventional methods—particularly the inability to capture transient or low-affinity interactions—offering a dynamic and high-resolution view of kinase signaling networks.

Application of Prob-PhI to MEK1 and MEK2, central regulators of the MAPK pathway, revealed distinct interactomes and phosphorylation substrates, uncovering novel signaling mechanisms. Although the endogenous kinases may compete for substrates and reduce labeling efficiency, our fold-change-based analysis relative to BASU-only controls ensures high-confidence identification of interactors. For substrate identification, the Prob-PhI workflow isolates biotinylated interactors before phosphopeptide enrichment, minimizing false positives even in the presence of endogenous competition. Notably, we identified LAMP3 as a MEK2-specific substrate phosphorylated at threonine 201, which promotes lysosomal activity. This finding exemplifies the platform’s ability to uncover kinase-specific functional relationships within complex cellular contexts.

Taken together, Prob-PhI provides a powerful and generalizable tool for dissecting kinase signaling networks with high specificity and temporal resolution. Its compatibility with diverse cell types and ability to resolve both interactomes and phosphoproteomes in a unified workflow make it well-suited for broad applications in cell signaling, drug discovery, and systems biology.

## Supplementary Material













## Notes

We have deposited the MS data archive to iProX, which is an official member of the Proteome Xchange Consortium. The raw and proceed data of MS spectrometry were deposited to iProX database under two subprojects. The data sets can be extracted through the links below, using the corresponding password, for the purpose of manuscript review. These links will remain valid for 360 days, starting from September 8, 2025. https://www.iprox.cn/page/DSV021.html;?url=1757293686922nE6v, password: pEnU https://www.iprox.cn/page/DSV021.html;?url=1757293806600A3WT, password: TQr6
Characterization of the MEK1 and MEK2 regulated proteome (Figure S1); phosphoproteins from its parental proteins (Figure S2) (PDF)
Quantitative proteomics dataset (Table S1) (XLSX)
MEK1 and MEK2 interactomes in HEK293T cells (Table S2) (XLSX)
Comprehensive phosphopeptide inventory (Table S3) (XLSX)
Cluster-specific phosphopeptide profiles (Table S4) (XLSX)
MEK1 and MEK2 interactomes in HeLa cells (Table S5) (XLSX)
