# Supplementary material for: Proximity-Based Phospho-Interactome (Prob-PhI) Characterization Reveals Distinct Signaling Activities of MEK1 and MEK2
Source: Anal Chem. 2026 Jan 29;98(5):3688–98. doi: 10.1021/acs.analchem.5c05557 (PMC12903061; doi:10.1021/acs.analchem.5c05557)
Supplement: Supplementary file 1 [file ac5c05557_si_002.pdf]

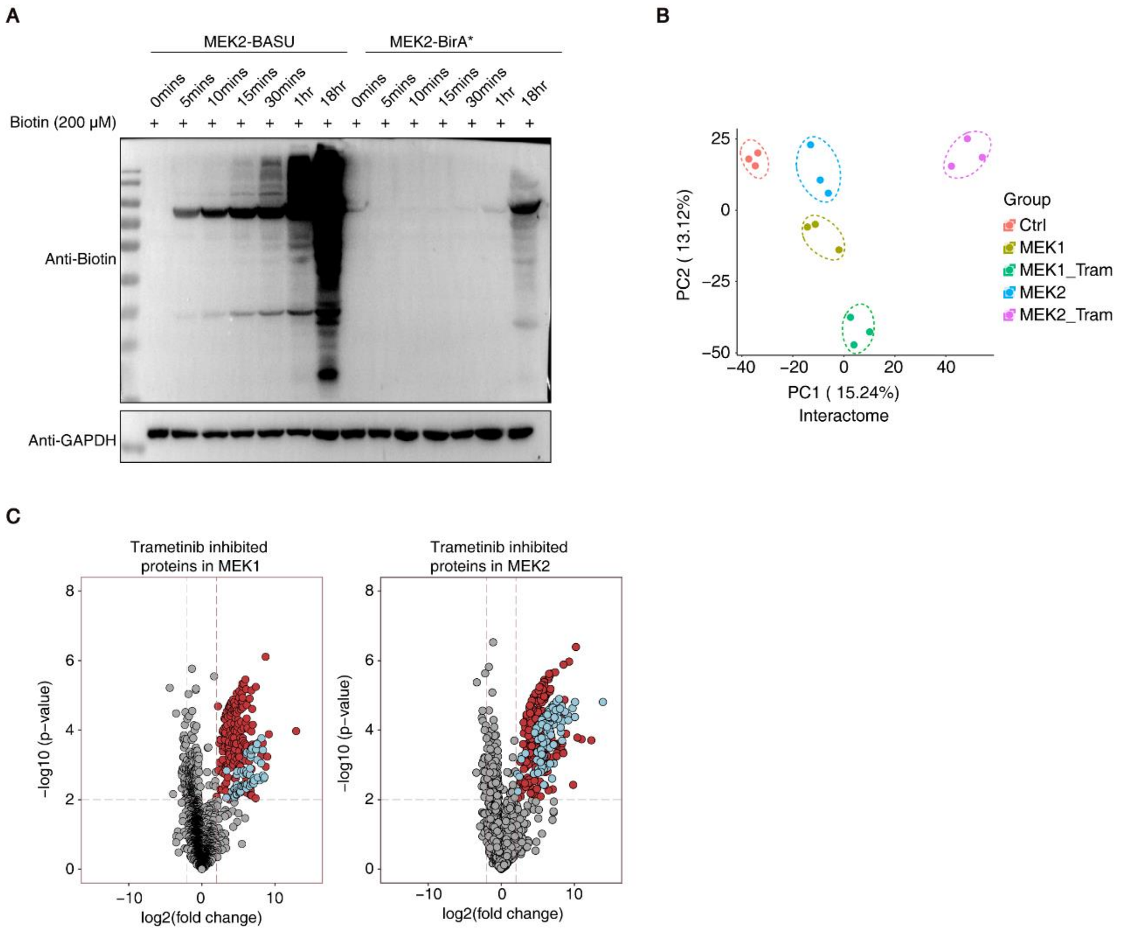

Supplemental Fig. S1 The characterization of the MEK1 and MEK2 regulated proteome.

(A) Western blot analysis of biotinylation time course experiments in HEK293T cells expressing MEK-BASU or MEK-BirA* fusion proteins. Cells were treated with biotin for the indicated times (0 mins, 5 mins, 10 mins, 15 mins, 30 mins, 1h, 18h) prior to lysis. Biotinylated proteins were detected using streptavidin-HRP.

(B) Principal component analysis (PCA) of protein interactome changes under different conditions. Different colors represent different treatment groups: Red circles indicate control group (Ctrl); Yellow circles indicate MEK1 expression group; Cyan circles indicate MEK1 expression group treated with trametinib; Light blue circles indicate MEK2 expression group; Purple circles indicate MEK2 expression group treated with trametinib.

(B) Volcano plot showing the effect of trametinib on proteins associated with MEK1 and MEK2, respectively. Red dots indicate significantly upregulated proteins by MEK1 expression, and blue dots indicate proteins that are significantly downregulated by trametinib. The thresholds for significance are log2FC >2 and p-value <0.01.

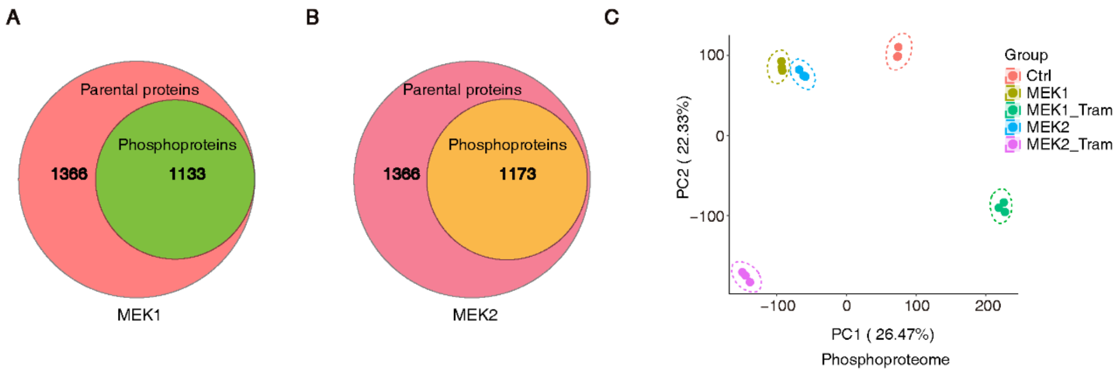

Supplemental Fig. S2 The phosphoproteins from its parental proteins.

(A) Venn diagram was used to illustrate the overlapping between the phosphoproteins identified for MEK1 and the proteins enriched in MEK1.

(B) Venn diagram was used to illustrate the overlapping between the phosphoproteins identified for MEK2 and the proteins enriched in MEK2.

(C) Principal component analysis (PCA) of phosphosites changes under different conditions. Different colors represent different treatment groups: Red circles indicate control group (Ctrl); Yellow circles indicate MEK1 expression group; Cyan circles indicate MEK1 expression group treated with trametinib; Light blue circles indicate MEK2 expression group; Purple circles indicate MEK2 expression group treated with trametinib.
